# Impact of laws prohibiting domestic violence on wasting in early childhood

**DOI:** 10.1371/journal.pone.0301224

**Published:** 2024-03-28

**Authors:** Pragya Bhuwania, Amy Raub, Aleta Sprague, Alfredo Martin, Bijetri Bose, Rachel Kidman, Arijit Nandi, Jere R. Behrman, Jody Heymann

**Affiliations:** 1 WORLD Policy Analysis Center, Fielding School of Public Health, University of California, Los Angeles, Los Angeles, California, United States of America; 2 Department of Family, Population and Preventive Medicine, Stony Brook University, Stony Brook, New York, United States of America; 3 Department of Epidemiology, Biostatistics and Occupational Health and the Institute for Health and Social Policy, Montreal, Quebec, Canada; 4 Department of Economics, University of Pennsylvania, Philadelphia, Pennsylvania, United States of America; University of Botswana, BOTSWANA

## Abstract

Intimate partner violence (IPV) affects an estimated 641 million women and girls globally with far-reaching consequences for the health of women and children. Yet, laws that prohibit domestic violence (DV) are not universal. Countries actively debate the effectiveness of DV laws in improving conditions given the inconclusive evidence on deterrent effects within households particularly in low- and middle-income countries that have limited infrastructure, and fewer resources to implement and enforce policy changes. This is the first study to rigorously examine the impact of DV laws on women’s health decision-making and the intergenerational impact on children’s wasting, a key predictor of mortality. We used the Demographic and Health Surveys (DHS) data collected between 2000–2020 across 23 African countries. Exploiting the staggered adoption of laws, we used a difference-in-differences study design to estimate the impact of DV laws in the treated countries compared to countries without such laws. We find that DV laws increased women’s decision-making autonomy in healthcare by 16.7% as well as other measures of women’s autonomy that matter for health such as financial autonomy by 6.3% and social mobility by 11.0%. The improvements in women’s autonomy translated into reductions in the probability of wasting among children aged 0–23 months by 5.4% points, a 30.9% reduction from the mean. DV laws also reduced wasting among older children aged 24–59 months by 3.6% points, a 38.7% reduction from the mean. The laws were effective in all 6 countries analyzed individually that criminalized DV. A civil prohibition in the seventh country was not found to be effective. The effect was positive and significant for all wealth and geographical categories. Our findings demonstrate the value of enacting criminal laws that prohibit domestic violence as one important tool to reducing the profound health impacts of IPV, a critical health and human rights issue.

## 1. Introduction

Domestic violence (DV) is both a human rights violation and a public health crisis. Globally, more than a quarter of married or partnered women aged 15–49 years report having experienced violence by spouses or partners in their lifetimes [1]. Women in Africa, the region of focus for this study, experience some of the highest rates of intimate partner violence (IPV). Moreover, statistics on IPV are likely underestimates of its prevalence, given the stigma still attached to disclosure and reporting. Within Africa, there is also immense variation across and within countries in estimated experiences of IPV [2, 3]. For example, one study estimated that more than half of women in Cameroon had experienced a form of IPV, compared to 9% of women in Comoros [2]. Levels of IPV have consistently been highest in central sub-Saharan Africa compared to other sub regions [3, 4].

IPV has consequences not only for women’s health [5–7], but also for the health of their children. A growing literature has linked maternal exposure to IPV with markers of childhood undernutrition including underweight and wasting, defined as low weight-for-height [8–10]. For example, a longitudinal study in South Africa found that exposure to emotional and physical IPV during pregnancy was associated with lower weight-for-age z-scores both at birth and at 12 months of age [11]. Similarly, a study of infants under 6 months old in 20 countries with among the highest burden of undernutrition found that infants of mothers exposed to physical IPV were 30% more likely to be wasted [12]. Moreover, one study in Ethiopia linked IPV directly to feeding practices, finding that children ages 6–23 months whose mother experienced IPV had 65% lower likelihoods of receiving minimum acceptable diets (defined as both receiving food from at least four of the seven food groups during the previous 24 hours and meeting minimum meal frequencies based on a scale tied to children’s ages and breastfeeding status) [13].

Through the Sustainable Development Goals (SDGs)—alongside a series of other human rights agreements dating back decades—countries around the world have committed to both ending IPV and markedly reducing wasting and other markers of child undernutrition [14]. Wasting is a particularly important early measure of undernutrition in childhood, disproportionately affecting children under two years of age. Furthermore, episodes of wasting contribute to risks of mortality and stunting (low height-for-age that results from chronic undernutrition) later in childhood with consequences for health that matter into adulthood [15–18]. Wasting indicates acute malnutrition and reflects recent episodes of infections and poor nutrition. Research shows that interventions are more effective at reducing wasting until age two than when children are older [19].

While exposure to domestic violence is shaped by many factors, including early marriage, educational attainment, and family resources, among others [20, 21], governments have important roles to play by legally prohibiting domestic violence and ensuring both the availability of protection for women who experience violence and penalties for perpetrators. Research suggests that domestic violence laws in low- and middle-income settings are associated with lower levels of violence. While not all associational studies have shown relationships [22]; the largest such study, a study of 54 LMICs found that having DV laws in place when a woman was married was associated with lower levels of emotional, physical, and sexual IPV [23]. A more rigorous study in Mexico that allows for causal inference by exploiting differences in policy over time and across states found that women living in states where DV laws were implemented experienced a 22% reduction in physical and sexual IPV compared to those in states without the laws [24]. However past studies have not examined the effects of these laws on children.

This is the first study to our knowledge to examine the impact of domestic violence laws on child outcomes in Africa. It expands on the existing literature to evaluate the impact of passing domestic violence laws on wasting in early childhood.

## 2. Methods

We employed a quasi-experimental research design to examine the impact of laws meant to protect women against domestic violence on their children’s wasting. Many countries in Africa adopted legal provisions to protect women against domestic violence since the early 2000s although many still lack such provisions. Exploiting the staggered adoption of legal provisions across the region, we used difference-in-differences study design (DD) to estimate the impact of prohibiting domestic violence on children’s wasting by comparing outcomes both within and across countries.

### 2.1. Data and sample

We linked an original legislative dataset on domestic violence laws to outcomes data collected by Demographic and Health Surveys (DHS) in Africa.

The longitudinal database on DV laws, created by the authors and the WORLD Policy Analysis Center at UCLA, spanned from 1990–2021. Legislative approaches were double-coded by policy researchers based on original legislative text. The data coding process fully accounted for longitudinal changes between 1990–2021 within all countries with respect to the following: (1) whether or not there exists a law against domestic violence, (2) whether or not there is a provision for restraining orders, and (3) whether or not domestic violence is considered a criminal offence. Countries with specific DV laws, broader laws prohibiting violence against women, or amendments to criminal codes to prohibit domestic violence and provide penalties were coded as having legal protections against domestic violence beginning the year these were first adopted. Countries with no legislations prohibiting DV or that do not provide recourse when violence occurs whether in the form of criminal penalties or access to restraining orders were coded as having no legal protections against DV during the study period. All countries in the study sample introduced one or more of these provisions all at once during the study period. There were no multiple or subsequent changes with respect to their adoption during the study period.

We obtained individual-level data on women and their children from DHS surveys harmonized by the Integrated Public Use Microdata Series (IPUMS) [25]. When a harmonized survey was not available, this was supplemented with DHS survey data [26] harmonized by a researcher from our research center following the IPUMS codebook to ensure consistency of methods across surveys. Our research did not require IRB approval because we used publicly available data. These surveys are nationally representative of women aged 15−49 years and collect detailed information on population health and demographic characteristics from female respondents aged 15−49 years in over 90 low- and middle-income countries. Standardized survey collection methodology and measurement techniques make DHS an appropriate data source for comparison across countries over time. We included all countries in Africa that have at least two DHS surveys with anthropometric data conducted between 2000 and 2021. This restriction was necessary to assess within-country changes over time in wasting.

The analytical sample pooled data from 70 DHS surveys across 23 countries that met our outcomes data restrictions. Table 1 shows the survey details and the year in which countries prohibited domestic violence. We identified 11 treated countries that have information on children born before and after the adoption of laws prohibiting domestic violence. The comparison group comprised 12 countries that either did not prohibit domestic violence or did so after the most recent available DHS survey. Guinea and Mozambique had only two years of data post law adoption. This was not sufficient time to determine treatment effects given that all laws take time to be implemented. Because of insufficient post-reform data, only unexposed birth cohorts in Guinea and Mozambique were used as part of the comparison group. Among 171,870 children aged 0–23 months, we excluded 4,896 (2.9%) cases with multiple births and 17,295 (10%) cases that had missing anthropometric measurements or implausible values of wasting that were flagged by DHS (weight-for-height z-scores below -5 standard deviation (SD) or above +5 SD). The final analytical sample included 149,679 children aged 0 to 23 months at the time of the interview.

**Table 1:** Mapping treated and comparison countries domestic violence legislative changes and DHS survey availability.

Country	DHS Surveys	Birth Cohort (min, max)	DV Law Year
**Treated countries (N = 11)**			
Benin	2001, 2006, 2011–2012, 2018	1999, 2018	2012
Ethiopia	2000, 2011, 2016	1999, 2016	2005
Ghana	2003, 2014	2001, 2014	2007
Lesotho	2004, 2009, 2014	2002, 2014	2010
Malawi	2000, 2004, 2010, 2016	1998, 2016	2006
Namibia	2000, 2007, 2013	1998, 2013	2003
Nigeria	2003, 2008, 2013, 2018	2001, 2018	2015
Rwanda	2000, 2005, 2010, 2014, 2020	1998, 2020	2008
Uganda	2001, 2006, 2016	1998, 2016	2010
Zambia	2002, 2007, 2014, 2018	1999, 2018	2011
Zimbabwe	2005, 2011, 2015	2003, 2015	2006
**Comparison countries (N = 12)**			
Burkina Faso	2003, 2010	2001, 2010	2018
Cameroon	2004, 2011, 2018	2002, 2018	NA
Chad	2004, 2015	2002, 2015	2017
DRC	2007, 2014	2005, 2014	NA
Egypt	2000, 2005, 2008, 2014	1998, 2014	NA
Guinea	2005, 2012, 2018	2003, 2016	2016
Kenya	2003, 2009, 2014	2001, 2014	2015
Liberia	2007, 2013	2005, 2013	2019
Mali	2001, 2006, 2013, 2018	1999, 2018	NA
Mozambique	2003, 2011	2001, 2009	2009
Niger	2006, 2012	2004, 2012	NA
Tanzania	2004, 2010, 2016	2002, 2016	NA

NOTE: Surveys were excluded that were conducted within two years of adoption of domestic violence laws because it is not possible, given the time needed for implementation, to clearly determine exposure to these changes and measure treatment effects. All surveys were obtained from IPUMS DHS except Cameroon 2018 and Rwanda 2020 that were directly obtained from the DHS website.

### 2.2. Measures

#### Outcome variables

We studied wasting as an indicator of undernutrition in children aged 0−23 months. Wasting, defined as low weight-for-height, usually indicates acute undernutrition. For all surveys in our sample, anthropometric measurements were collected for children under age 5. DHS personnel measured weights for all children under 5, lengths in a recumbent position for children under 2, heights in a standing position for children over 2, and ages in months at the time of measurements. The difference between children’s values from the median of a well-nourished reference population of the same age and sex was expressed in standard deviations using the 2006 WHO Child Growth Standards. Children with weight-for-height scores below −2 standard deviations from the reference standard were considered wasted.

Literature suggests that wasting is more likely to occur in the first two years of life [18], and this is consistent with our analysis of the distribution of wasting across different age groups. Our primary analysis focuses on wasting among children aged 0−23 months where wasting is most likely to occur and is more predictive of future health and wellbeing. In addition, we also examine the impact of these laws on slightly older children aged 24−59 months as part of our secondary analysis.

#### Domestic violence laws and exposure variable

The exposure of interest in our study was whether women were legally protected against DV at the time their child was born. These protections were in the form of laws prohibiting domestic violence and having access to recourse in the form of criminal penalties or restraining orders. Because we examine the impact on children and because an individual woman may have children both before and after a domestic violence law is passed, we defined exposure at the level of an individual child who was considered exposed to these legal protections if they were born after the law was adopted and unexposed otherwise. This definition ensures access to protections all the way from infancy through early childhood. Table 2 shows the sample sizes and the prevalence of wasting in exposed and unexposed categories for each country in the sample.

**Table 2:** Sample size and sample characteristics of children aged 0–23 months in the treated and comparison countries.

Country	DV Law			Wasting (N)		Wasting (%)	
	(N) Not exposed	(N) Exposed	(%) Exposed	Not exposed	Exposed	Not exposed	Exposed
**Treated**							
Benin	7,182	4,976	40.9	1,020	364	14.2	7.3
Ethiopia	1,370	6,773	83.2	252	1,056	18.4	15.6
Ghana	627	1,021	62.0	91	82	14.5	8.0
Lesotho	1,049	560	34.8	77	31	7.3	5.5
Malawi	4,715	3,529	42.8	425	186	9.0	5.3
Namibia	591	1,827	75.6	61	186	10.3	10.2
Nigeria	16,078	4,589	22.2	3,238	462	20.1	10.1
Rwanda	1,733	4,086	70.2	149	127	8.6	3.1
Uganda	1,867	1,778	48.8	165	109	8.8	6.1
Zambia	3,161	7,512	70.4	254	483	8.0	6.4
Zimbabwe	1,205	3,787	75.9	102	196	8.5	5.2
All treated	39,578	40,438	50.5	5,834	3,282	17.5	10.3
**Comparison**							
Burkina Faso	4,030	-	-	1,095	-	27.2	-
Cameroon	4,810	-	-	345	-	7.2	-
Chad	4,803	-	-	973	-	20.3	-
DRC	4,010	-	-	439	-	11.0	-
Egypt	13,680	-	-	1,232	-	9.0	-
Guinea	2,217	-	-	299	-	13.5	-
Kenya	9,928	-	-	654	-	6.6	-
Liberia	2,536	-	-	278	-	11.0	-
Mali	10,203	-	-	1,794	-	17.6	-
Mozambique	2,704	-	-	188	-	7.0	-
Niger	2,740	-	-	581	-	21.2	-
Tanzania	8,002	-	-	581	-	7.2	-
All comparison	69,663	-	-	8,459	-	9.7	-

#### Control variables

We included potential determinants of childhood wasting from the DHS based on existing literature on Africa [27–30]. Individual-level characteristics included the children’s sex, ages in months, birth orders, mothers’ ages at the time of births, and mothers’ education. Household-level characteristics included wealth, total members in the household, and urban/rural residences. We also included potential country-level confounding factors such as GDP per capita growth (annual %), domestic governmental health expenditure (% of GDP), and level of urbanization at the time of the survey obtained from the World Bank’s World Development Indicators database. Please see S1 Table for distributions of covariates within treated and comparison countries.

### 2.3. Statistical analyses

#### Effects of laws prohibiting domestic violence

We used two-way fixed-effects regressions (TWFE) to estimate the effects of laws prohibiting domestic violence on the probabilities of wasting among children. Building on the simpler two-group two-time period difference-in-differences (DD) estimator, TWFE allows for multiple groups that change over multiple periods, making it a popular tool for estimating the effects of policy changes on outcomes. Many studies have used these methods to estimate the impacts of policies rolled out over time [31–33]. This strategy estimates a weighted average treatment effect across locations and time periods by controlling for location-specific and period-specific shocks [34]. We used the following regression model (Eq 1) to estimate the effects of DV laws,

$${Probit(Y}_{ict})=\beta_{0}+\beta_{1}{DV\_law}_{ict}+\delta X_{ict}+\xi X_{ct}+\gamma_{c}+\delta_{t}+e_{ict}$$ (1)

where *Y_ict_* was a binary variable indicating whether child *i* born in country *c* in year *t* was wasted and *DV_law_ict_* was a dummy variable that captures exposure to a DV law (it takes the value 1 for all children born in the post period in treated countries and 0 otherwise). The main coefficient of interest was *β*_1_ that estimates the effect of prohibiting domestic violence in treated countries in comparison to countries without any legal protections. *X_ict_* represented individual- and household-level characteristics. The model accounted for time-invariant differences among countries as well as shared secular time trends over the study period across countries using country (*γ_c_*) and birth-year (*δ_t_*) fixed effects. Finally, *X_ct_* controlled for potential time-varying country-level confounders.

The probit model was used to analyze the binary indicators of wasting among children. We calculated marginal effects at means using post-estimation commands to report absolute differences in the probabilities of wasting. All models accounted for individual-level de-normalized sampling weights as recommended by the DHS for carrying-out multi-survey pooled analysis [35] and incorporated robust standard errors clustered at the country level. We conducted all analyses in STATA 14.2.

After estimating the effect of DV laws on the pooled sample of all treated and control countries, we also estimated country-specific impacts. We compared each treated country one at a time to the pooled data from all comparison countries using the same estimation strategy. We do not perform separate analysis for four out of the ten treated countries that did not have sufficient sample size either during the post-reform period (Ghana and Lesotho) or pre-reform period (Namibia and Zimbabwe) for carrying out individual country-specific analysis. An added advantage of separate country-specific regressions is that they help circumvent one of the main limitations of TWFE models with staggered treatment (discussed further under model assumptions). With only one treated country at a time in the analytical sample, the exposed cohorts are not compared to those already treated. Instead, the treatment effects are estimated by purely comparing the treated to the untreated over the study period [36].

Lastly, we also estimated the impact of DV laws on children aged 24−59 months. While the prevalence of wasting is generally lower among children in this age group, DV laws could still impact them. We tested the impact of the policy separately on children who were exposed to the policy for a part of their life(interviewed after the reforms but born within five years preceding the change) and children who were exposed to the policy during the entire course of their life (interviewed and born after the reforms).

#### Examining model assumptions

The validity of DD estimates relies upon the assumption that the trends in prevalence of outcome measures in the treated countries would have been similar to the trends in the comparison countries had the treated countries not adopted DV laws. We tested this assumption in two ways. First, we plotted the prevalence of wasting for all treated countries against all comparison countries among children born between 1998, the first birth cohort of children surveyed in the sample, up to 2003, the last year before any of the birth cohorts were exposed to DV laws in the treated countries. Second, we formally tested this assumption by regressing the outcomes on a country-level dummy variable, indicating treated or comparison, interacted with the linear time trend during the pre-policy period. The significance of the interaction term indicated whether the trends in the prevalence of wasting were similar across treated and comparison countries before DV laws were adopted.

More recent literature on TWFE models demonstrates that a causal interpretation of the TWFE DD estimates requires both a parallel-trends assumption and homogenous treatment effects that are constant over time [37]. TWFE DD estimators that exploit the variation in treatment timing across multiple units equal a weighted average of all possible two-group/two-period DD estimators in the data. Negative weights may arise when already-treated units act as controls [34, 37, 38]. We conducted additional tests to address these concerns. We carried-out event-study DD analysis with leads up to the year of reform and lags after the reform to show clear post-treatment changes. The resulting event-study graph demonstrates the change in treatment effects over time.

Another common threat to the validity of difference-in-differences estimation models is whether other changes coincided with the intervention in the treatment countries during the study period that might have impacted the outcome of interest. We address this concern in several ways. First, we mitigate the risk of other concurrent changes driving the results by including many treatment countries in the pooled sample and by also testing them individually. We compare children born before and after DV laws in the treatment countries to those born during the same time in the comparison countries. It is unlikely for other policy shifts to coincide with DV laws in multiple treatment countries. Second, we rerun the analysis on a narrower study time frame of 7 years pre- and post-adoption of DV laws to reduce the chances of picking up other changes in the treatment countries. Third, we inspect and analyze the timing of passage of laws related to broader child rights including provisions for prohibiting violence against children to evaluate the possibility of confounding of the impact estimates. Fourth, we re-estimate the model after controlling for country-specific linear time trends to account for other factors that influence children’s wasting and that vary within a country over time [39, 40].

#### Sensitivity analysis

We tested the sensitivity of our estimates in several ways. First, children born just before countries prohibited domestic violence could have also been impacted by these changes for a part of their early childhood. By including them in the analysis as not exposed to the policy, treatment effects could be underestimated if DV laws impacted children born before the time of law adoption. We tested the robustness of our results to this potential misclassification by dropping children born within two years preceding the adoption of DV laws from the analysis. We also tested the impact of the policy on this group of children by creating a separate exposure category of its own alongside that of the fully exposed children and compared these groups to those not exposed to the policy.

Second, many countries had small sample sizes for a few birth years. These birth years might be less reliable because fewer children contributed to the calculation of average levels of wasting. We dropped country-specific birth years that had fewer than 50 children to test the sensitivity of the results to low samples sizes.

Third, we re-estimated the pooled estimate without the four countries that had insufficient data for country-specific analysis to check the sensitivity of the coefficient estimates to the exclusion of these countries from the sample. Fourth, we visually inspected the trends in wasting prevalence for each country. We also examined country-specific birth cohorts that had an absolute change of more than 20 percentage points in wasting prevalence relative to the year just before or after. We identified spikes in wasting prevalence in 3 of the 23 study countries and excluded the birth cohorts with these spikes to test whether any data anomalies or country-specific external shocks influenced the pooled estimates.

Fifth, TWFE DD estimates could be biased in the presence of negative weights. We examined whether the data resulted in any negative weights and ran additional sensitivity tests to address related concerns. We reviewed the source of negative weights and assessed their likely severity by re-running the pooled analysis on truncated data without country-birth years with negative weights [34].

Sixth, we controlled for partner’s age and education to further improve the precision of our coefficient estimate. We are unable to control for partner’s alcohol consumption, a recognized risk factor for exposure to domestic violence [41, 42] because of lack of sufficient data; however, this should not affect the accuracy of the estimates of the policy impact unless this changed at the same time as the domestic violence laws.

Seventh, while we tested the parallel-trends assumption between the treated and comparison group, we also examined whether the results were sensitive to any of the individual countries that comprised the comparison group. We dropped one comparison country at a time from the analytical sample and reran the regressions to check if the results changed.

#### Heterogeneity analyses

The impact of DV laws may vary with household background characteristics. Women from poor households and those residing in rural areas could have limited access to information on DV laws and have difficulties utilizing the legal means to fight violence. We examined whether DV laws impacted children differentially depending on their household socio-economic status (SES) and urban/rural residence. DHS separates all interviewed households into five wealth quintiles based on household asset ownership. We interacted policy exposure with wealth quintiles to determine how SES affects the relationship between domestic violence laws and childhood wasting. Similarly, we interacted urban/rural residence with policy exposure to estimate the differential impact on children living in urban versus rural areas.

#### Exploring mechanisms

DV laws might impact childhood wasting through many channels. We explored one such pathway related to women’s autonomy in household decision-making. A large body of literature shows that women’s autonomy has important consequences not only for maternal health but also for the health of their children [43–46]. More financially autonomous women are more likely to have control over resources that they can allocate to improving the health of their children. Women with greater decision-making power on matters related to healthcare can better utilize important services such as antenatal and postnatal care, thereby impacting maternal and child health. Women’s social autonomy can also influence children’s health by giving mothers better access to advice and support from family or relatives.

DHS collects information on whether women had final say in decisions regarding spending their own earnings, visiting family or relatives, and women’s healthcare. We coded these decision-making variables as 1 if women had a final say alone or along with partner or someone else in the household and 0 otherwise. We used the same regression model shown in Eq 1 to estimate the impact of protections against domestic violence on women’s autonomy in household decision-making.

## 3. Results

### 3.1. Impact of DV laws on wasting among children aged 0–23 months

Table 3 reports the marginal effects (difference in predicted probabilities in absolute percentage points) from the probit TWFE model shown in Eq (1). These estimates measure the impact of prohibiting domestic violence on the probabilities of wasting when compared to countries without such laws. As evident from the pooled estimate, DV laws reduced the probabilities of wasting among children aged 0−23 months by 5.4 percentage points on average, a 30.9% reduction from the mean.

**Table 3:** Impact of laws that protect women against domestic violence on the probability of wasting among their children aged 0–23 months.

Country	Sample Size	Wasting
		Treatment effects^†^ (95% CI)
		
**Pooled**	146,136	−5.4***
		(−7.6, −3.3)
Benin	73,384	−3.8***
		(−6.4, −1.2)
Ethiopia	69,373	−9.3***
		(−11.8, −6.8)
Malawi	69,474	−6.0***
		(−7.7, −4.3)
Nigeria	81,888	−4.1*
		(−8.6, 0.3)
Rwanda	67,046	−5.9***
		(−7.5, −4.4)
Uganda	64,873	−5.2***
		(−6.9, −3.5)
Zambia	71,890	0.6
		(−1.8, 2.9)

^†^Treatment effects reported the change in the probability of wasting in percentage points terms with 95% confidence intervals (CI)

*** p<0.01

** p<0.05

* p<0.1

Standard errors clustered at the country-level

TWFE model with birth year fixed effects and country fixed effects.

Controls: Child’s age in months, child’s sex, child’s birth order, mother’s age at the time of birth, mother’s education, family size, wealth, rural/urban residence, country’s GDP per capita growth rate, domestic public expenditure on health (as % of GDP), and country’s level of urbanization.

Table 3 also shows the country-specific impacts of prohibiting domestic violence on the probabilities of wasting. DV laws reduced the probabilities of wasting in 6 countries — Benin, Ethiopia, Malawi, Nigeria, Rwanda, and Uganda. The highest declines were observed in Ethiopia where wasting was reduced by 9.3 percentage points on average. The lowest declines were observed in Benin where wasting was reduced by 3.8 percentage points on average. For Malawi, Nigeria, Rwanda, and Uganda, the impact on wasting ranged from 4−6 percentage points. DV laws did not seem to impact wasting in only one country — Zambia.

### 3.2. Model assumptions

**Fig 1:**
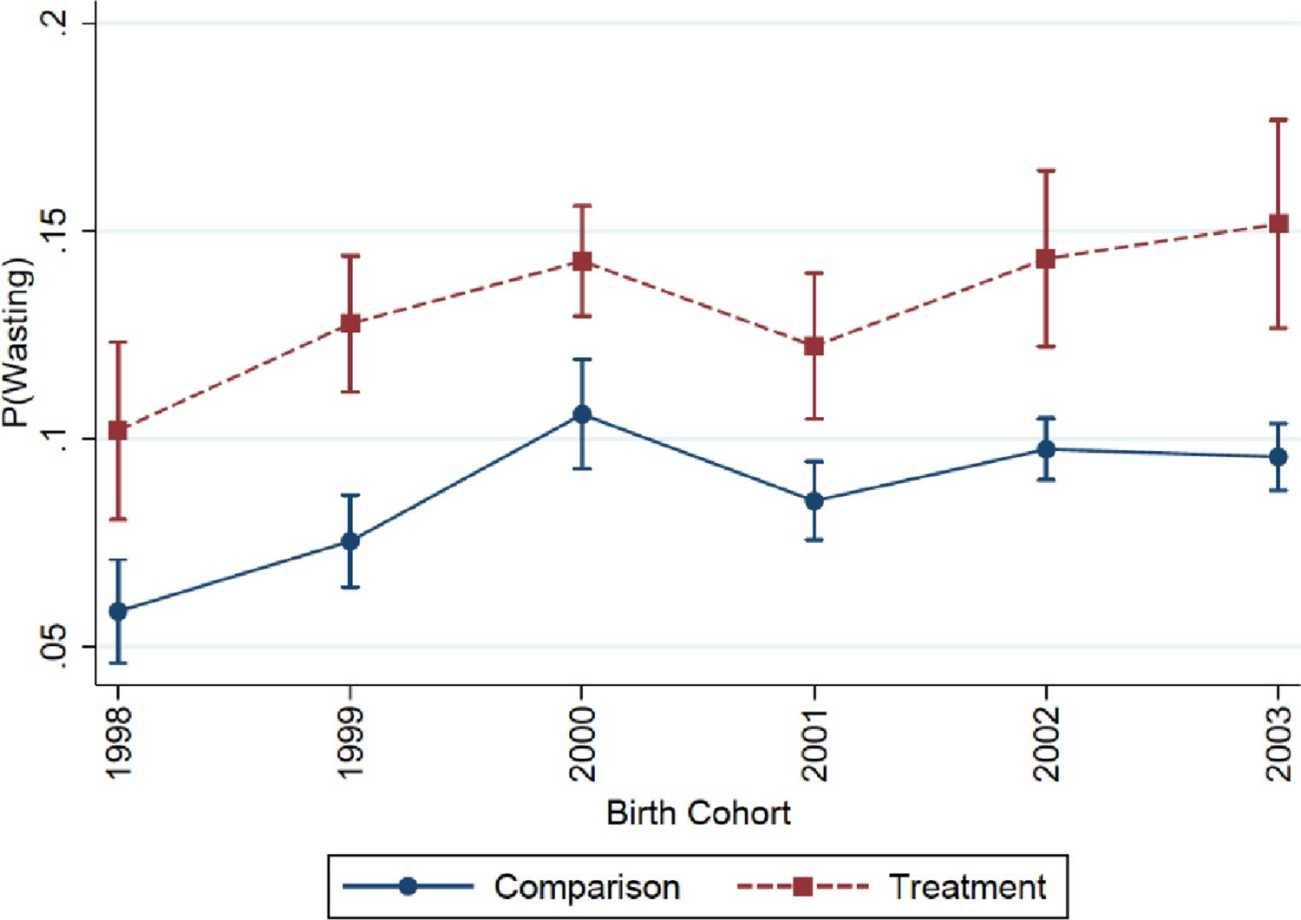
Trends in the prevalence of wasting among children aged 0–23 months before the adoption of domestic violence laws. There was no statistically significant difference in the pre-trends.

**Fig 2:**
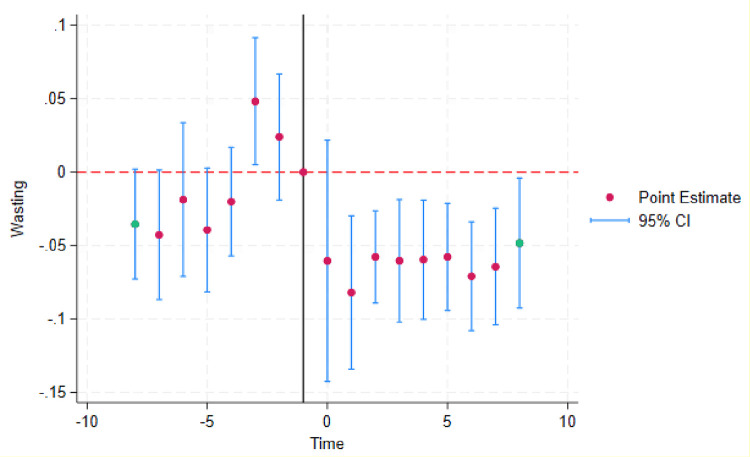
Event-study estimates of the effect of domestic violence laws on childhood wasting. The graph shows the difference between the pre- and post-reform periods between the treated and comparison countries. The cohorts are centered at the last untreated cohort that acts as the reference category and the bands show 95% confidence intervals. The post-reform period is suggestive of homogenous treatment effects over time and the pre-reform period suggests no major differences in the treated and comparison countries before the passage of laws.

Prior to the adoption of DV laws, the trends in the prevalence of wasting were similar in the treated and comparison countries. The coefficient of the interaction term (–0.1, 95% CI [–4.8, 4.7]) between treatment status and the linear time trend during the pre-policy years was not significant for wasting, indicating no evidence for differing trends between the treated and comparison countries before DV laws were beginning to be adopted. Fig 1 shows the plot of marginal effects (predicted probabilities) of wasting by treatment status during the pre-policy years. It is evident that while the treated countries had a higher baseline level of wasting than the comparison countries, the trends in the prevalence of wasting were remarkably similar across the two groups. We present the event-study plot in Fig 2 that shows no evidence for heterogenous treatments effects varying over time. The post-reform period estimates are similar over the years after DV laws were adopted.

We address concerns relating to other competing explanations for the impact of DV laws on children’s wasting in Table 4. Panel A shows the results using a narrower study period of ±7 years of changes in DV laws in the treatment countries. The impact was slightly reduced to an estimated effect of –4.8 percentage points. We also checked whether or not the treatment countries introduced broader child rights laws including prohibiting violence against children during the study period (please see S2 Table). Of the 11 treatment countries, Benin and Lesotho were the only countries that adopted child rights laws so close to the DV laws during the study period that we are unable to parse out their impacts separately. We re-estimate the pooled analysis after dropping these countries from the sample. Malawi and Rwanda adopted child rights laws during the study period but we can separate out the impact of DV laws by dropping Malawi 2016 and Rwanda 2000 surveys from the analytical sample.

**Table 4:** Impact of DV laws on the probability of wasting among their children aged 0–23 months after accounting for other concurrent changes during the study period.

Sample	Sample Size	Wasting Treatment effects^†^ (95% CI)
Panel A *(narrower study time frame)*	131,379	−4.8*** (−6.6, −2.9)
Panel B *(accounting for child rights laws)*		
Pooled	129,559	−5.1*** (−7.7, −2.6)
Malawi	67,497	−5.8*** (−7.3, −4.3)
Rwanda	66,209	−4.9*** (−6.5, −3.3)
Panel C (*country-specific linear time trend*)	146,136	−7.9*** (−11.4, −4.4)

^†^Treatment effects reported the change in the probability of wasting in percentage points terms with 95% confidence intervals (CI)

*** p<0.01

** p<0.05

* p<0.1

Standard errors clustered at the country-level

TWFE model with birth year fixed effects and country fixed effects.

Controls: Child’s age in months, child’s sex, child’s birth order, mother’s age at the time of birth, mother’s education, family size, wealth, rural/urban residence, country’s GDP per capita growth rate, domestic public expenditure on health (as % of GDP), and country’s level of urbanization.

Panel B shows the updated pooled and individual impact estimates of DV laws after accounting for these changes. Our estimates remain stable and we can conclude that we are indeed capturing the impact of DV laws on children’s wasting during the study period. Lastly, Panel C shows the magnitude of the pooled estimate further increases after controlling for country-specific linear time trends, suggesting that the effect of DV laws is not confounded.

### 3.3. Sensitivity analysis

We report the sensitivity of our estimated effects to alternative exposure specifications in Table 5. In the estimates discussed to this point, we defined children as exposed to these legal protections if they had access to protections all the way from infancy through early childhood. We now show the robustness of this approach by addressing the potential misclassification of children exposed to these laws during only a part of their lives. Panel A shows the results from models using a restricted sample that drops children born within two years preceding the legal changes. The results remained remarkably stable, suggesting no impact on children born during the pre- period and lending support to the robustness of our specification. We corroborated these results in Panel B where we estimated the impact of reforms on two separate exposure categories of children by comparing them to children not exposed to the reforms. Panel B shows that young children born right before the reforms had no change in the probabilities of wasting whereas children born afterward showed declines in the probabilities of wasting by 5.4 percentage points on average as compared to children who were not exposed.

**Table 5:** Sensitivity analyses showing effects of domestic violence laws on the probability of wasting among children aged 0–23 months at the time of interview.

Sample	Sample Size	Wasting Treatment effects^†^ (95% CI)
Panel A *(dropping children born within two years preceding the change)*	143,676	−5.4*** (−7.6, −3.3)
Panel B *(two exposure categories)*	146,136	
born within 2 years preceding the change		0.9 (−4.1, 5.8)
born after the policy change		−5.4*** (−7.5, −3.2)
Panel C *(dropping birth cohorts with sample size less than 50)*	145,579	−5.3*** (−7.5, −3.1)
Panel D *(dropping four countries with insufficient sample size for individual analysis)*	135,471	−5.7*** (−8.1, −3.3)
Panel E *(dropping birth cohorts with implausible shocks in wasting prevalence)*	145,178	−5.4*** (−7.6, −3.2)
Panel F *(dropping country-years with negative weights)*	139,352	−4.6*** (−6.5, −2.7)
Panel G (*controlling for partner’s age and education*)	129,067	−5.6*** (−7.9, −3.4)
Panel H *(dropping one comparison country at a time)*		
Burkina Faso	142,107	−5.5*** (−7.6, −3.3)
Cameroon	141,327	−5.9*** (−8.3, −3.4)
Chad	141,340	−5.5*** (−7.6, −3.4)
DRC	142,134	−5.7*** (−7.7, −3.7)
Egypt	132,466	−5.0*** (−7.5, −2.4)
Guinea	143,919	−5.4*** (−7.6, −3.2)
Kenya	136,208	−5.6*** (−7.9, −3.3)
Liberia	143,602	−5.4*** (−7.6, −3.3)
Mali	139,413	−5.7*** (−7.9, −3.4)
Mozambique	143,432	−5.5*** (−7.6, −3.3)
Niger	143,399	−5.3*** (−7.5, −3.1)
Tanzania	138,134	−5.7*** (−8.4, −3.0)

^†^Treatment effects reported the change in the probability of wasting in percentage points terms with 95% confidence intervals (CI)

*** p<0.01, ** p<0.05, * p<0.1

Standard errors clustered at the country-level

TWFE model with birth year fixed effects and country fixed effects.

Controls: Child’s age in months, child’s sex, child’s birth order, mother’s age at the time of birth, mother’s education, family size, wealth, rural/urban residence, country’s GDP per capita growth rate, domestic public expenditure on health (as % of GDP), and country’s level of urbanization.

Panel C shows that the results were also robust to low sample sizes — the coefficient estimates hardly changed from 5.4 to 5.3 when we dropped country-specific birth years that had fewer than 50 children. In Panel D, the pooled estimate was slightly higher than the original estimate when we excluded treated countries with insufficient data for estimating country-specific effects. In Panel E, we found the pooled estimate to be robust to huge shocks in the prevalence of wasting due to data anomalies or external country-specific events. We also showed the robustness of the treatment effect estimate to the presence of negative weights in Panel F. We found a few negative weights in later birth years (see S1 Fig). As suggested by [34], we truncated the data by dropping these country-year observations and reported the results from the re-estimated model as an additional sensitivity check. The coefficient estimate reduced slightly to 4.6 percentage points but remained statistically significant, suggesting that the problem of negative weights was not severe.

In Panel G, we show that controlling for partner’s characteristics such as age and education does not change the treatment effect. Lastly, Panel H shows the coefficient estimates from regressions that dropped one comparison country at a time and once again we find that the results were not particularly sensitive to any individual comparison country. The estimates ranged from 5.0 to 5.9 percentage points. As a final robustness check, we estimated Eq (1) using linear probability models (LPM) and the results remained the same (see S3 Table for results).

### 3.4. Impact on children aged 24–59 months

In Table 6, we report the impact of DV laws on children aged 24–59 months. We obtained marginal effects from a similar probit TWFE model as shown in Eq (1) where we interacted the policy variable with the two types of exposure categories of children—partially exposed (born before DV laws but measured after) and fully exposed (born and measured after DV laws). We find that even though the impact was not as strong as that for children aged 0–23 months, these reforms reduced the probability of wasting among children aged 24–59 months. DV laws reduced the probability of wasting by 1.6 percentage points among children who were exposed to the laws during a part of their life and by 3.6 percentage points among children who were fully exposed to the laws during their entire life course. The parallel trends model assumption was satisfied in that the trends in the prevalence of wasting among children aged 24–59 months were similar in treated and comparison countries during the pre-reform period (see S2 Fig).

**Table 6:** Impact of laws that protect women against domestic violence on the probability of wasting among their children aged 24–59 months.

Treatment	Wasting
	Treatment effects^†^ (95% CI)
Domestic-violence laws	
*exposed partially*	−1.6*
	(−3.3, 0.1)
*exposed fully*	−3.6***
	(−5.8, −1.4)
Observations	181,838

^†^Treatment effects reported the change in the probability of wasting in percentage points terms with 95% confidence intervals (CI)

*** p<0.01

** p<0.05

* p<0.1

Standard errors clustered at the country-level

TWFE model with birth year fixed effects and country fixed effects.

Controls: Child’s age in months, child’s sex, child’s birth order, mother’s age at the time of birth, mother’s education, family size, wealth, rural/urban residence, country’s GDP per capita growth rate, domestic public expenditure on health (as % of GDP), and country’s level of urbanization.

### 3.5. Heterogeneity analyses

Table 7 shows DV laws reduced childhood wasting across all wealth categories and urban/rural residents. However, the impact was the smallest among the bottom wealth quintile children at −4.2 percentage points and the largest among the top wealth quintile children at −7.0 percentage points. The impact among the middle categories ranged from −5.2 to −5.6 percentage points. Furthermore, the impact in urban areas was stronger at −6.2 percentage points versus rural areas at −5.1 percentage points.

**Table 7:** Differential impact of laws that protect women against domestic violence on the probability of wasting among their children aged 0–23 months across various household wealth categories and urban/rural residence.

Household characteristics	Wasting
	Treatment effects^†^ (95% CI)
Household wealth	
*Poorest*	−4.2***
	(−6.6, −1.7)
*Poor*	−5.4***
	(−8.1, −2.7)
*Middle*	−5.6***
	(−7.4, −3.8)
*Rich*	−5.2***
	(−7.8, −2.6)
*Richest*	−7.0***
	(−9.7, −4.4)
Household residence	
*Urban*	−6.2***
	(−8.9, −3.4)
*Rural*	−5.1***
	(−7.0, −3.2)
Observations	146,136

^†^Treatment effects reported the change in the probability of wasting in percentage points terms with 95% confidence intervals (CI)

*** p<0.01, ** p<0.05, * p<0.1

Standard errors clustered at the country-level

TWFE model with birth year fixed effects and country fixed effects.

Controls: Child’s age in months, child’s sex, child’s birth order, mother’s age at the time of birth, mother’s education, family size, wealth, rural/urban residence, country’s GDP per capita growth rate, domestic public expenditure on health (as % of GDP), and country’s level of urbanization.

### 3.6. Impact of DV laws on women’s decision-making autonomy

We report the impact of DV laws on women’s autonomy in household decision-making in Table 8. These are marginal effects from the probit TWFE model as shown in Eq (1) where the outcome is now a binary variable indicating whether or not a woman has a say in various aspects of household decision-making. We find that protecting women against domestic violence increased the probability of women’s final say in decisions related to spending their own earnings by 5.3 percentage points, visiting family or relatives by 6.3 percentage points, and their health care by 5.5 percentage points. These results suggest that the reductions in children’s wasting as a result of the passage of DV laws might have followed from these laws improving women’s autonomy within the household. We did not find evidence for any major difference in decision-making trends in the treated and comparison countries before DV laws were adopted, thereby satisfying the parallel trends model assumption (see S3 Fig).

**Table 8:** Impact of laws that protect women against domestic violence on the probability of women having a final say in household decision-making.

Exposure to DV laws	Sample size	Women’s household decision-making Treatment effects^†^
		(95% CI)
Women’s final say in		
*Spending their own earnings*	253,777	5.3***
		(2.6, 8.0)
*Visiting family or relatives*	582,811	6.3**
		(0.2, 12.3)
*Women’s health care*	579,712	5.5*
		(−0.3, 11.3)

^†^Treatment effects reported the change in the probability of women having a say in household decision-making in percentage points terms with 95% confidence intervals (CI)

*** p<0.01

** p<0.05

* p<0.1

Standard errors clustered at the country-level

TWFE model with survey year fixed effects and country fixed effects.

Observations for women’s say in spending their own earning was lower because this question was asked only to those women who reported being paid in cash or kind during the previous year

## 4. Discussion

This study uses a quasi-experimental design to evaluate the impact of laws prohibiting domestic violence on wasting in early childhood. We found that passing DV laws reduced wasting in young children. On average, the enactment of a DV law was associated with a 5.4 percentage-point decline in wasting among children ages 0–23 months, a 30.9% reduction from the mean. These findings make an important contribution to the literature on domestic violence, providing causal evidence to support the role of legislation to reduce IPV. The use of difference-in-difference techniques allowed us to separate the timing of the policy changes from other factors that might have been influencing child wasting within and across countries. By including a large number of treated countries, we also reduced the risk of simultaneous policy changes that might contaminate the results. A series of sensitivity checks further bolster the confidence in the estimated effects.

Domestic violence laws were effective at reducing wasting among children across all wealth categories living in urban and rural areas. While the laws had a greater impact on children from households in the top wealth quintile and those living in urban areas, the effect was significant across the board. Furthermore, DV laws also reduced wasting among older children aged 24–59 months. The results supported our hypothesis that even though the greatest impact occurs during the first two years of life, all children under 5 years benefited from these laws.

While there are multiple mechanisms through which DV laws might reduce childhood wasting, we provide evidence in favor of one important mechanism related to women’s autonomy in household decision-making. We found that women protected against domestic violence had greater financial, social, and healthcare autonomy. These findings suggest that DV laws might be reducing wasting among children by providing their mothers with greater access to resources and decision-making power within the household.

The study has several limitations worth noting. First, we could not examine the effect of DV laws on women’s experiences of violence. Direct experience of violence is not only important for its own sake but also for its potential intergenerational impacts. However, even though DHS has extensive information on women’s lifetime experience of violence, the data on violence within the past year is scant. The limited data do not allow for testing of parallel trends before the passage of laws which is a critical model assumption in DID analysis.

Second, while we explored plausible pathways related to changes in women’s autonomy in household decision-making, it was beyond the scope of this study to examine all pathways through which protections against domestic violence might impact child undernutrition. In addition to reduction in the actual prevalence of domestic violence within households with young children, other pathways such as changes in social, cultural, and gender norms, changing nutritional status of pregnant and breastfeeding women, or greater women’s economic participation due to protections against domestic violence could have been at play as well.

Our third limitation relates to the extent to which we can code and analyze various aspects of domestic violence laws. We do not have enough data to statistically parse out the impacts of specifics of these laws with respect to definitions of domestic violence, existence of restraining orders, or nature of criminal penalties. Our results, however, do suggest that criminalizing domestic violence might be critical given that the only individual country to not show any impact is the one that does not criminalize domestic violence. Fourth, an important study limitation is that we could not measure the quality of implementation of these changes across countries. It was beyond the scope of this study to gather information on budget allocation, provision and access to support services and response centers, and convictions and prosecutions under these laws. The variation in the country-specific results may be due to important differences in implementation.

Fifth, even though the presence of negative weights could be concerning, the robustness checks provide evidence for their lack of severity. Furthermore, Jakiela [34] suggests that when identifying assumptions seem plausible, traditional TWFE models offer advantage over a range of newer alternative estimators that may have less statistical power than the pooled estimator. Lastly, even though the results are likely to be generalizable because we studied a large number of treated and comparison countries within the African region, effect sizes may be different in other geographies for a variety of reasons.

As countries consider how to meet their international commitments in the SDGs, this study provides new evidence about the role of national policy approaches in creating intergenerational change at scale. Passing laws prohibiting IPV is fundamental to countries’ commitments to eliminate all forms of violence against women (SDG 5.2) and to ensure that their legal frameworks promote gender equality (SDG 5.1.1). Our findings that these laws also help reduce child wasting demonstrate that they likewise have a powerful role in achieving the SDG targets to reduce child undernutrition (SDG 2.2). As policymakers consider different approaches for improving nutrition in early childhood, this study demonstrates that laws banning IPV can have significant long-term impacts and may represent a critical complementary approach to more immediate interventions like targeted feeding programs. Future research should extend this work to examine the impact of laws prohibiting domestic violence on stunting, which has also been linked to maternal exposure to IPV [47–49]. While eliminating IPV and ending child undernutrition are human rights imperatives, achieving these goals could also powerfully support economic growth. Previous studies have estimated that continued inaction on IPV costs sub-Saharan African countries $2 billion from the economic impact that stunting has on earnings in adulthood [44]. This study provides further rigorous support for the pathway between legislative change and economic growth.

We found variation in the effect of DV laws across countries in our study. In 6 countries, domestic-violence laws were associated with reductions in wasting, which ranged from 3.8 percentage points in Benin to 9.3 percentage points in Ethiopia. Zambia was the only country where a DV law did not seem to impact childhood wasting. Notably, Zambia is the only study country that has a law against domestic violence but does not criminalize it. Domestic violence matters in rural Zambia are settled in local or customary courts where there is no jurisdiction of criminal recourse. This is inconsistent with broad recommendations by the UN [50]. In addition, even though DV law was passed in the national assembly in Nigeria, not all states adopted these laws at the state level. This might help explain the relatively smaller coefficient size with lower statistical significance for reductions in wasting in Nigeria. We also investigated other aspects of DV laws such as whether they were gender-specific or had direct provisions for children. Benin was the only treatment country to adopt a DV law specific to women and girls. Moreover, only 2 countries, Benin and Namibia, also banned violence against dependents or children. Further research is needed to understand the role of additional aspects of DV laws that might affect implementation, incidence of violence, and recourse that ultimately impact the wellbeing of women and their children.

Variation in findings across countries is not surprising given expected variation across countries in implementing DV laws, as well as the heterogeneity in prior studies linking nutritional outcomes to maternal experiences of IPV and the heterogeneity across countries in contexts. Future research should examine more deeply the factors that lead to effective implementation in countries, and how these laws impact other measures of child and maternal health and well-being. Literature suggests that domestic violence laws may offer the greatest benefit to women from households with higher levels of education or household wealth. Findings from this study indicate that domestic violence laws may also be reaching women in households with lower levels of family resources, given that socioeconomic status is closely associated with child undernutrition. More research is needed to understand where and how these policies are effectively reaching the most vulnerable women. Future research should also examine how societal norms as well as complementary legislations, such as laws and policies that support girls’ educational attainment and child marriage laws, impact child health and nutrition.

## Supporting information

S1 Table Sample distribution of covariates in the treatment and comparison countries. (DOCX)

S2 Table Timing of the adoption of DV laws and child rights laws in the treatment countries. (DOCX)

S3 Table Impact of laws that protect women against domestic violence on the probability of wasting among their children aged 0–23 months using Linear Probability Models (LPM). (DOCX)

S1 Fig Weights used for calculating the weighted average of the TWFE DD estimate. Grey squares show untreated units, yellow squares show treated units with positive weight, and purple shows country-year combinations with negative weights. We excluded birth cohorts with negative weights that are marked in purple from the pooled analysis to assess the sensitivity of treatment effect estimate to negative weights.
(TIF)

S2 Fig Trends in the prevalence of wasting among children aged 24–59 months prior to the adoption of domestic violence laws. There was no statistically significant difference in the pre-trends.
(TIF)

S3 Fig Trends in women’s autonomy in household decision-making in treated and comparison countries prior to the adoption of domestic violence laws. There was no statistically significant difference in the pre-trends.
(TIF)
