# Supplementary material for: Impact of laws prohibiting domestic violence on wasting in early childhood
Source: PLoS One. 2024 Mar 28;19(3):e0301224. doi: 10.1371/journal.pone.0301224 (PMC10977741; doi:10.1371/journal.pone.0301224)
Supplement: S1 Table — (DOCX) [file pone.0301224.s001.docx]

S1 Table: Sample distribution of covariates in the treatment and comparison countries

| Country | Age | Sex  (Boy)  % | Birth order | Mother’s age at birth | Mother’s education  years | Household members  (N) | Urban % | GDP pc growth  rate | Public health expenditure |
| --- | --- | --- | --- | --- | --- | --- | --- | --- | --- |
| **Treatment** |  |  |  |  |  |  |  |  |  |
| Benin | 11.3 | 49.5 | 3.0 | 25.7 | 2.0 | 6.9 | 37.8 | -0.1 | 0.7 |
| Ethiopia | 11.0 | 50.0 | 3.3 | 25.7 | 2.1 | 5.7 | 18.5 | 7.3 | 1.1 |
| Ghana | 11.4 | 50.1 | 2.6 | 25.8 | 5.2 | 5.5 | 35.9 | 3.8 | 1.4 |
| Lesotho | 11.0 | 47.2 | 2.0 | 23.5 | 7.5 | 6.4 | 18.0 | 3.9 | 4.3 |
| Malawi | 11.6 | 49.6 | 2.5 | 23.2 | 5.1 | 5.2 | 26.9 | 2.0 | 1.9 |
| Namibia | 11.4 | 50.7 | 2.0 | 23.3 | 7.7 | 7.2 | 36.2 | 1.6 | 4.4 |
| Nigeria | 11.1 | 49.9 | 3.3 | 26.0 | 5.5 | 6.6 | 33.7 | 2.0 | 0.6 |
| Rwanda | 11.3 | 50.7 | 2.6 | 26.6 | 4.6 | 5.0 | 20.5 | 3.2 | 2.0 |
| Uganda | 11.4 | 50.9 | 3.1 | 24.0 | 5.3 | 5.9 | 18.8 | 1.4 | 1.6 |
| Zambia | 11.6 | 49.7 | 3.1 | 25.0 | 6.1 | 6.3 | 32.2 | 2.8 | 1.8 |
| Zimbabwe | 11.1 | 50.0 | 2.4 | 24.7 | 8.8 | 5.8 | 28.1 | 2.7 | 3.9 |
| All treatment | 11.3 | 49.9 | 3.0 | 25.2 | 4.9 | 6.2 | 28.5 | 2.5 | 1.5 |
| **Comparison** |  |  |  |  |  |  |  |  |  |
| Burkina Faso | 11.1 | 49.9 | 2.8 | 24.1 | 1.2 | 7.9 | 21.1 | 0.6 | 1.5 |
| Cameroon | 11.3 | 50.3 | 3.1 | 24.8 | 5.7 | 7.9 | 41.1 | 0.9 | 0.4 |
| Chad | 10.5 | 50.0 | 3.8 | 24.9 | 1.8 | 7.1 | 27.4 | 4.2 | 1.3 |
| DRC | 11.1 | 50.7 | 3.4 | 25.6 | 5.0 | 6.6 | 32.7 | 3.2 | 0.5 |
| Egypt | 11.5 | 51.4 | 2.2 | 24.7 | 8.0 | 6.0 | 36.7 | 2.2 | 1.5 |
| Guinea | 12.1 | 51.5 | 3.1 | 24.9 | 1.5 | 8.2 | 27.9 | 2.9 | 0.5 |
| Kenya | 11.5 | 50.9 | 3.0 | 25.2 | 6.7 | 5.7 | 30.4 | 1.2 | 1.8 |
| Liberia | 11.1 | 52.5 | 2.9 | 24.3 | 3.0 | 6.8 | 34.2 | 4.7 | 0.8 |
| Mali | 11.0 | 50.7 | 3.3 | 24.4 | 1.4 | 6.8 | 26.9 | 1.3 | 1.2 |
| Mozambique | 12.6 | 49.3 | 2.4 | 22.4 | 3.0 | 6.7 | 37.8 | 5.7 | 1.4 |
| Niger | 10.7 | 49.2 | 3.7 | 25.2 | 1.1 | 7.8 | 23.9 | 0.4 | 2.0 |
| Tanzania | 11.4 | 49.5 | 3.0 | 25.1 | 5.5 | 7.1 | 21.2 | 3.2 | 1.4 |
| All comparison | 11.3 | 50.6 | 2.9 | 24.7 | 4.6 | 6.8 | 30.3 | 2.2 | 1.3 |

NOTE: Children’s age reported in months, Domestic public health expenditure reported as % of GDP
