# Supplementary material for: Impact of laws prohibiting domestic violence on wasting in early childhood
Source: PLoS One. 2024 Mar 28;19(3):e0301224. doi: 10.1371/journal.pone.0301224 (PMC10977741; doi:10.1371/journal.pone.0301224)
Supplement: S2 Table — (DOCX) [file pone.0301224.s002.docx]

S2 table: Timing of the adoption of DV laws and Child Rights laws in the treatment countries

| Country | DHS Surveys | DV Law Year | Child Rights Law Year |
| --- | --- | --- | --- |
| Benin* | 2001, 2006, 2011-2012, 2018 | 2012 | 2015 |
| Ethiopia | 2000, 2011, 2016 | 2005 | 1957 |
| Ghana | 2003, 2014 | 2007 | 1998 |
| Lesotho* | 2004, 2009, 2014 | 2010 | 2011 |
| Malawi^†^ | 2000, 2004, 2010, 2016 | 2006 | 2010 |
| Namibia | 2000, 2007, 2013 | 2003 | 2015 |
| Nigeria | 2003, 2008, 2013, 2018 | 2015 | 2003 |
| Rwanda^†^ | 2000, 2005, 2010, 2014, 2020 | 2008 | 2001 |
| Uganda | 2001, 2006, 2016 | 2010 | 1997 |
| Zambia | 2002, 2007, 2014, 2018 | 2011 | 2022 |
| Zimbabwe | 2005, 2011, 2015 | 2006 | 1972 |

Of the 11 treatment countries, 7 adopted child rights laws outside of the study period

* Benin and Lesotho are the only countries that adopted Child Rights laws so close to the DV laws during the study period that we are unable to parse out their impacts separately

^†^ Malawi and Rwanda adopted Child Rights laws during the study period but we can separate out the impact of DV laws by dropping Malawi 2016 and Rwanda 2000 surveys from the analytical sample
