# Supplementary material for: Impact of laws prohibiting domestic violence on wasting in early childhood
Source: PLoS One. 2024 Mar 28;19(3):e0301224. doi: 10.1371/journal.pone.0301224 (PMC10977741; doi:10.1371/journal.pone.0301224)
Supplement: S3 Table — (DOCX) [file pone.0301224.s003.docx]

Table S3: Impact of laws that protect women against domestic violence on the probability of wasting among their children aged 0–23 months using Linear Probability Models (LPM)

| Country | Sample Size | Wasting |
| --- | --- | --- |
|  |  | Treatment effects^†^  (95% CI) |
|  |  |  |
| **Pooled** | 146,136 | −6.1*** |
|  |  | (−8.9, −3.3) |
| Benin | 73,384 | −5.3*** |
|  |  | (−8.6, −1.9) |
| Ethiopia | 69,373 | −11.6*** |
|  |  | (−16.5, −6.8) |
| Malawi | 69,474 | −6.5*** |
|  |  | (−10.5, −2.5) |
| Nigeria | 81,888 | −6.2** |
|  |  | (−10.6, −1.7) |
| Rwanda | 67,046 | −5.7*** |
|  |  | (−8.8, −2.6) |
| Uganda | 64,873 | −6.3*** |
|  |  | (−9.1, −3.5) |
| Zambia | 71,890 | 0.1 |
|  |  | (−1.7, 3.8) |

^†^Treatment effects reported the change in the probability of wasting in percentage points terms with 95% confidence intervals (CI) using Linear Probability Models (LPM)

*** p<0.01, ** p<0.05, * p<0.1

Standard errors clustered at the country-level

TWFE model with birth year fixed effects and country fixed effects.

Controls: Child’s age in months, child’s sex, child’s birth order, mother’s age at the time of birth, mother’s education, family size, wealth, rural/urban residence, country’s GDP per capita growth rate, domestic public expenditure on health (as % of GDP), and country’s level of urbanization.
