# Supplementary figures and images for: Impact of laws prohibiting domestic violence on wasting in early childhood
Source: PLoS One. 2024 Mar 28;19(3):e0301224. doi: 10.1371/journal.pone.0301224 (PMC10977741; doi:10.1371/journal.pone.0301224)

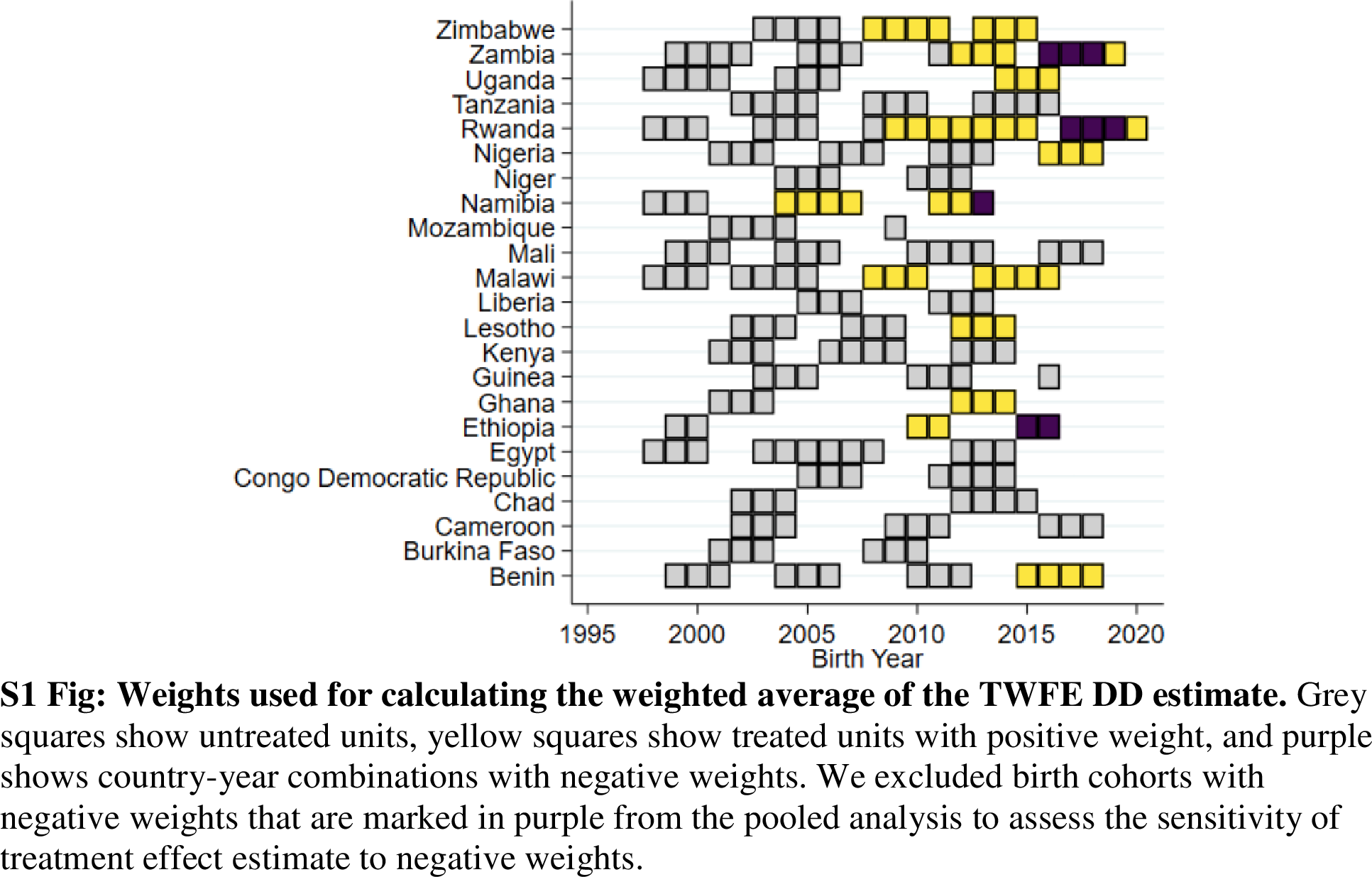

Supplement: S1 Fig — Grey squares show untreated units, yellow squares show treated units with positive weight, and purple shows country-year combinations with negative weights. We excluded birth cohorts with negative weights that are marked in purple from the pooled analysis to assess the sensitivity of treatment effect estimate to negative weights. (TIF) [file pone.0301224.s004.tif]

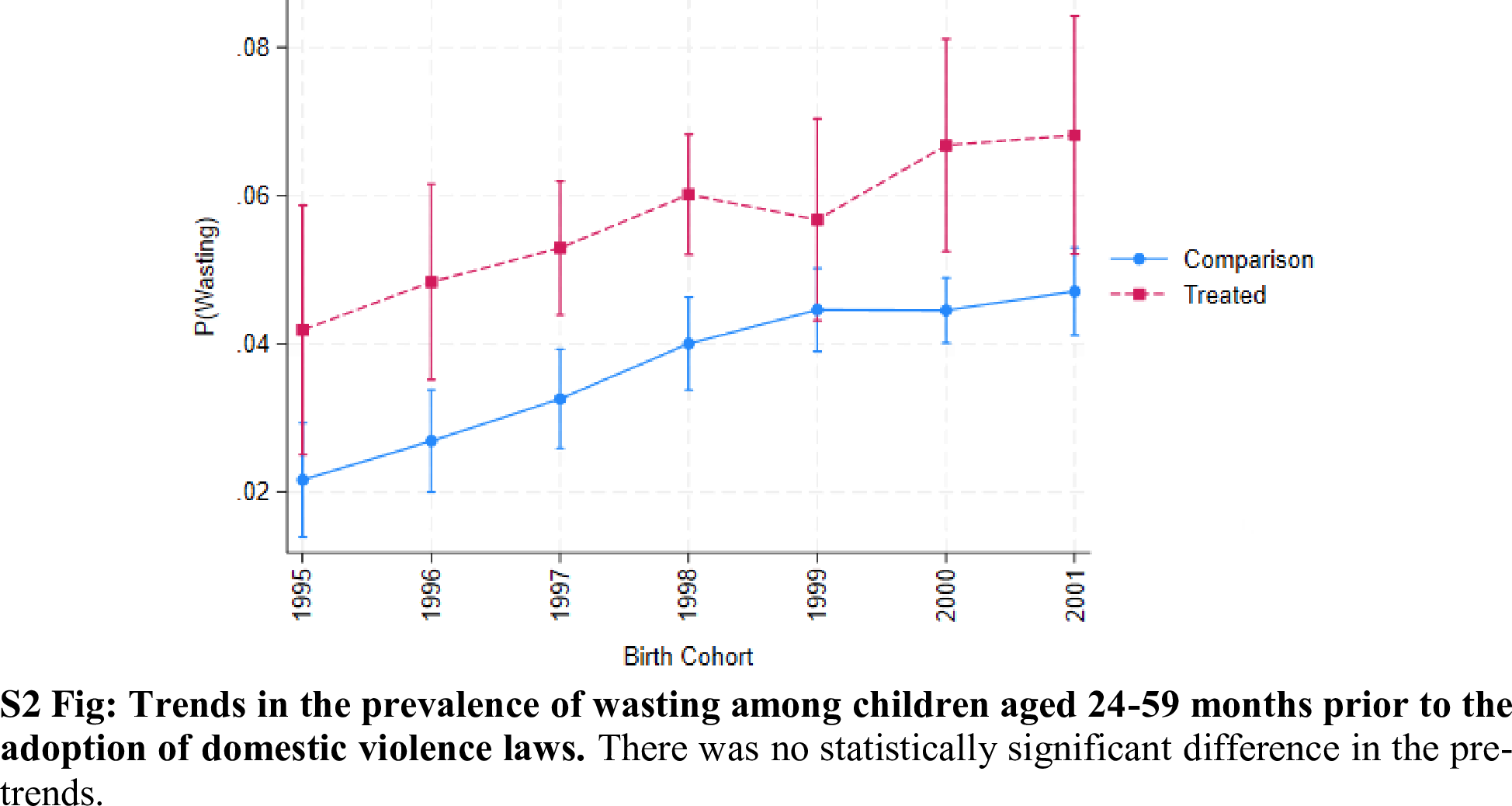

Supplement: S2 Fig — There was no statistically significant difference in the pre-trends. (TIF) [file pone.0301224.s005.tif]

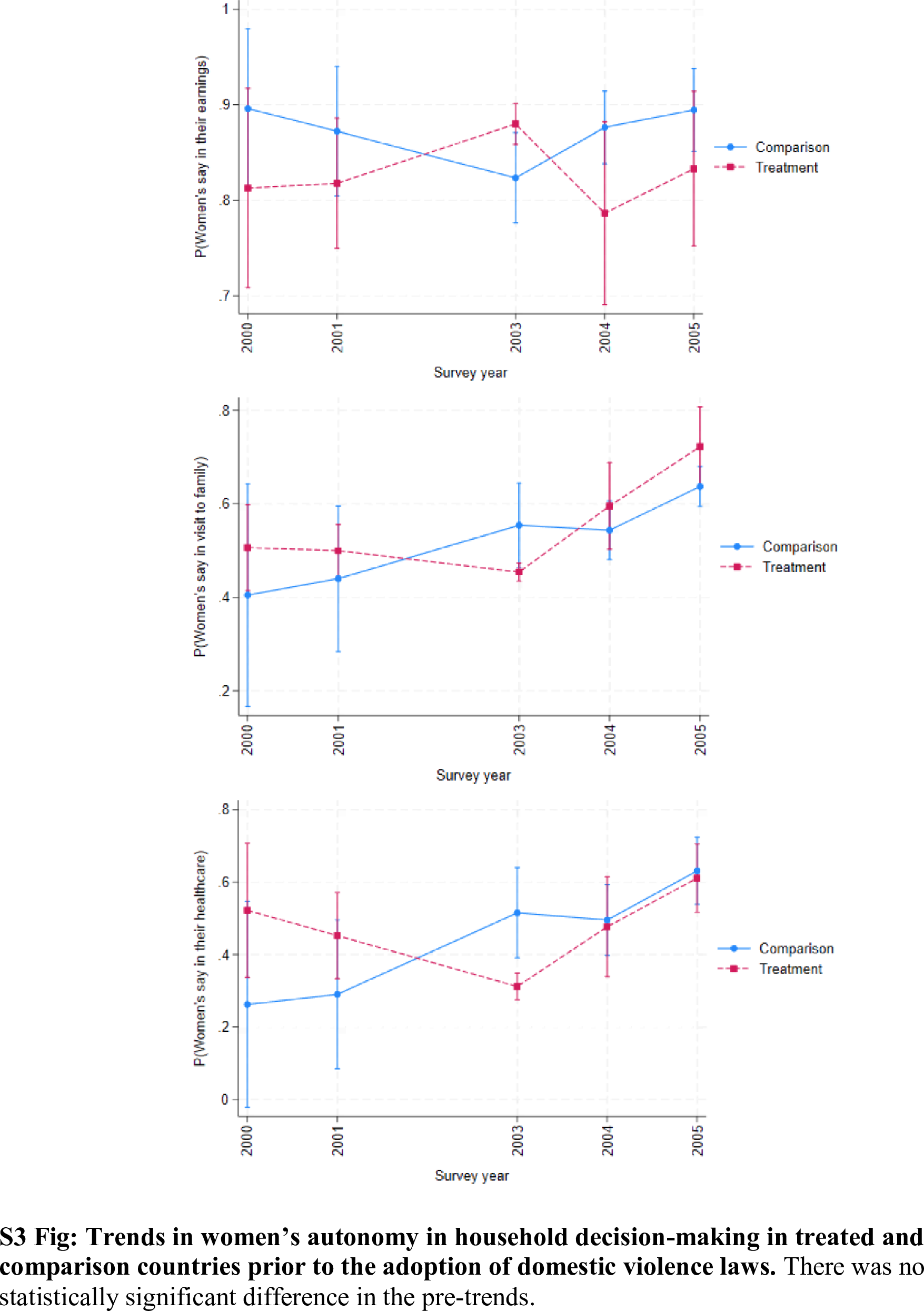

Supplement: S3 Fig — There was no statistically significant difference in the pre-trends. (TIF) [file pone.0301224.s006.tif]
